# Inhibitors of *Mycobacterium marinum* virulence identified in a *Dictyostelium discoideum* host model

**DOI:** 10.1371/journal.pone.0181121

**Published:** 2017-07-20

**Authors:** Hajer Ouertatani-Sakouhi, Sébastien Kicka, Gianpaolo Chiriano, Christopher F. Harrison, Hubert Hilbi, Leonardo Scapozza, Thierry Soldati, Pierre Cosson

**Affiliations:** 1 Faculty of Medicine, Department of Cell Physiology and Metabolism, University of Geneva, Geneva, Switzerland; 2 Department of Biochemistry, University of Geneva, Geneva, Switzerland; 3 Pharmaceutical biochemistry, School of pharmaceutical sciences, University of Geneva, University of Lausanne, 30 Quai Ernst Ansermet, Geneva, Switzerland; 4 Max von Pettenkofer Institute, Department of Medicine, Ludwig-Maximilians University Munich, Munich, Germany; 5 Institute of Medical Microbiology, Department of Medicine, University of Zürich, Gloriastrasse 30/32, Zürich, Switzerland; Institut de Pharmacologie et de Biologie Structurale, FRANCE

## Abstract

Tuberculosis remains one of the major threats to public health worldwide. Given the prevalence of multi drug resistance (MDR) in *Mycobacterium tuberculosis* strains, there is a strong need to develop new anti-mycobacterial drugs with modes of action distinct from classical antibiotics. Inhibitors of mycobacterial virulence might target new molecular processes and may represent a potential new therapeutic alternative. In this study, we used a *Dictyostelium discoideum* host model to assess virulence of *Mycobacterium marinum* and to identify compounds inhibiting mycobacterial virulence. Among 9995 chemical compounds, we selected 12 inhibitors of mycobacterial virulence that do not inhibit mycobacterial growth in synthetic medium. Further analyses revealed that 8 of them perturbed functions requiring an intact mycobacterial cell wall such as sliding motility, bacterial aggregation or cell wall permeability. Chemical analogs of two compounds were analyzed. Chemical modifications altered concomitantly their effect on sliding motility and on mycobacterial virulence, suggesting that the alteration of the mycobacterial cell wall caused the loss of virulence. We characterized further one of the selected compounds and found that it inhibited the ability of mycobacteria to replicate in infected cells. Together these results identify new antimycobacterial compounds that represent new tools to unravel the molecular mechanisms controlling mycobacterial pathogenicity. The isolation of compounds with anti-virulence activity is the first step towards developing new antibacterial treatments.

## Introduction

Tuberculosis (TB) caused by *Mycobacterium tuberculosis* represents a threat to public health worldwide. One third of the world population is infected and TB accounts for 1.8 million yearly deaths (WHO Global tuberculosis report 2016). Antibacterial TB treatments such as isoniazid, rifampicin, pyrazinamide and ethambutol have been used for decades to treat TB. Multi drug resistance (MDR) to these conventional drugs has emerged worldwide [1].

Efforts are currently made to develop novel antimycobacterial drugs, and this requires a better understanding of the biology of mycobacterial infections and the identification of new drug targets. Novel antibiotics have proven extremely difficult to discover in the last decades [2]. A promising alternative may be to identify compounds that inhibit bacterial virulence that could be used either in combination with or instead of antibiotics [3].

Researchers have used different bacteria to study mycobacterial infection, including *M*. *smegmatis*, *M*. *bovis* and *M*. *marinum*. *M*. *marinum* is the closest genetic relative of the *M*. *tuberculosis* complex [4] and causes TB-like infections in fish [5]. Eighty-five percent of *M*. *marinum* loci encoding putative virulence genes have homologous genes in *M*. *tuberculosis*. Thus, due to its relative safety and similar pathogenicity, *M*. *marinum* is widely used as a reliable model to study mycobacterial infections.

Similarly, free-living amoebae such as *Acanthamoeba castellanii* or *Dictyostelium discoideum* provide cost-effective and ethically unproblematic models to measure bacterial virulence and to screen for anti-virulence compounds [6–8]. *Dictyostelium discoideum* amoebae have proven a valuable non-mammalian host to study bacterial virulence and host resistance with human pathogens such as *Legionella*, *Klebsiella*, *Mycobacteria* [9–11], *Pseudomonas* (reviewed in [12, 13]), *Vibrio cholera* [14], and *Salmonella typhimurium* [15]. This system has also been used to identify compounds inhibiting bacterial infectivity [6, 8, 16].

Here, we used a *Dictyostelium*-*M*. *marinum* infection model to identify new chemical compounds inhibiting mycobacterial virulence. Preliminary characterization of the compounds suggests that they inhibit a variety of virulence mechanisms. A significant group of compounds affects functions requiring an intact mycobacterial cell wall.

## Materials and methods

### Cell culture

*Dictyostelium discoideum* strain DH1–10 [17] was grown at 21°C in HL5 medium and subcultured twice a week to maintain a maximal density of 10^6^ cells ml^−1^. The parental *M*. *marinum* M strain (referred to as wild-type (WT) for simplicity) and the RD1 mutant were gift from Pr. L. Ramakrishnan [18]. It was cultured in Middlebrook 7H9 (Difco) supplemented with 10% OADC (Becton Dickinson), 0.5% glycerol (Sigma Aldrich), 0.05% Tween 80 (Sigma Aldrich) at 30°C in shaking culture. The *M*. *marinum* TesA mutant [19] was a gift from Dr. L. Kremer (Montpellier University, CNRS, France). *M*. *marinum* FadD28 [20] was a gift from Pr. J. Liu (University of Toronto, Canada). The *M*. *marinum* strain used to measure intracellular replication carries the pMV306-lux plasmid [11, 21]. *Klebsiella pneumoniae* is a previously described non-pathogenic laboratory isolate and was grown in LB (lysogeny broth) medium [22].

### Growth of *Dictyostelium* on bacteria

*M*. *marinum* virulence was measured as previously described [19]. Briefly, 10 ml of mid-log phase mycobacterial cultures were centrifuged for 5 min at 2,000 rpm, resuspended in 5 ml of an overnight culture of *K*. *pneumoniae* diluted to 10^−5^ in LB medium, and residual clumps were disrupted by passaging through a 25-gauge blunt needle. In each well of a 24-well plate, 50 μl of the bacterial suspension were plated on 2 ml of solid SM (standard medium)-agar medium supplemented with glucose [11] and left to dry for 2–3 h. Finally, 1,000 *Dictyostelium* cells were added in the center of the well. Plates were incubated for 5–9 days at 25°C and the formation of phagocytic plaques was monitored visually. To test the effect of a compound on *M*. *marinum* virulence, it was added to the SM-Agar medium at 30 μM (6 μl of DMSO in 2 ml of SM-Agar) and allowed to diffuse in the agar for 1 h before the addition of bacteria. Except during the first test screens (that led to the identification of the M4 compound), a negative control (Bacteria+Dictyostelium+DMSO), and a positive control (Bacteria+Dictyostelium+M4 30μM) were included in every plate.

### Chemical compound collections

Different collections of chemical compounds were used for the screening. We initially screened a library of 1,040 compounds compiled by the NINDS: the NIH Custom Collection for FDA-approved drugs and bioactive compounds. Then, we screened the Prokinase library composed of 1,035 compounds targeting cellular kinases (http://www.proteinkinase-research.org/), and the open access malaria box collection of 400 hit and lead candidates targeting malaria (http://www.mmv.org/research-development/open-access-malaria-box. In addition, we tested a targeted library enriched in putative anti-virulence compounds that we designed specifically for this project (1,600 compounds, Kicka et al, “in preparation”). Finally, we tested a set of 6,000 compounds from the commercially available highly diverse Maybridge collection (Table 1). Selected compounds are identified by their ZINC number (zinc.docking.org) or their CAS number (pubchem.ncbi.nlm.hih.gov).

**Table 1:** Chemical libraries screened in this study.

	Library	Compounds	Primary hits	Confirmed hits	Hit rate (%)
Targeted	Sinergia	1,260	68	15	1.15
	Prokinase	1,200	24	9	0.75
Diverse	NINDS	1,099	15	7	0.6
	Malaria Box	400	38	6	1.5
	Maybridge	6,000	171	11	0.18

### Antibiotic assay

In order to test the inhibitory effect of compounds on mycobacterial growth in a 24-well plate, each molecule was added to a well containing 2 ml of 7H11 agar medium at the indicated concentration ranging from 0.3 μM to 30 μM. Then, 1,000 bacteria were deposited in each well and plates were incubated at 30°C for 7 days to allow bacterial growth. The minimal inhibitory concentration was determined visually and represented the minimal concentration at which even a minor inhibition of mycobacterial growth was detected. A similar assay was used to measure antibiotic effects of compounds on *K*. *pneumoniae*, except that LB medium was used instead of 7H11very similar results were determined in 3 independent experiments. For a few compounds, the absence of antibiotic effect on mycobacteria was verified in 7H9 liquid medium by measuring optical density of the suspension at 600nm, and by counting colony-forming units after plating of diluted aliquots on 7H11 medium.

### “Sliding motility” on soft agar medium

“Sliding motility” was visualized essentially as previously described [23]. Briefly, 7H9 medium (5 ml) supplemented with 0.3% agarose was poured in each well of a 6 well plate. Compounds were added at the indicated concentration (30 to 0.3 μM). With a toothpick one colony was inoculated in the center of the well and plates were covered and incubated at 30°C for 10 days. Sliding motility, i.e. the ability of the mycobacteria to spread over the agarose surface was determined visually.

### Bacterial aggregation and permeability

We used flow cytometry to determine the effect of compounds on bacterial aggregation and permeability. Bacteria were cultured in the presence of the indicated compounds for 48 h. They were then washed, resuspended in 500 μl of 50 mM potassium phosphate buffer (pH 7) and incubated at room temperature in the presence of 6 μM ethidium bromide for 20 min. Forward scatter and fluorescence intensity were measured by flow cytometry. Fluorescence due to dye accumulation within bacteria was determined at an excitation and emission wavelength of 545 nm and 600 nm, respectively. Forward scatter provided a measure of the aggregation status of bacteria. Fluorescence entry was measured for bacteria with the same levels of forward scatter, and provided a measure for bacterial cell permeability.

### Infection of *Dictyostelium* with luminescent *M*. *marinum*

As described previously [11], mycobacteria expressing luciferase [21] were grown for 24 h in the presence of antivirulence compounds (10 μM) in shaking (220 rpm) 6-well plates in 5 ml of 7H9 medium containing 10% OADC (Becton Dickinson), 0.5% glycerol (Sigma Aldrich) and 0.02% tyloxapol (Sigma Aldrich). The cultures were then washed twice with HL5c medium and passed through a 25-gauge blunt needle to disrupt residual clumps, then added onto 10 cm dishes containing adherent *Dictyostelium* cells (around 5 × 10^7^) at a multiplicity of infection (MOI) of 10:1. Dishes were centrifuged at 500 x *g* for 10 min in a Beckman Coulter Allegra 6R centrifuge, turned 180 degrees, and centrifuged a second time to avoid accumulation of cells and bacteria in one side of the dish. The cells were left at 25°C for an additional 10–20 min. Then, excess extracellular bacteria were carefully removed by washing 4–5 times with 10 ml of HL5c without detaching *Dictyostelium* cells. Amikacin (10 μM) was added to inhibit extracellular proliferation of bacteria [24, 25]. The infected cells were then detached and added to a 96-well plate (White F96 MicroWell™ plates, non-treated (Nunc)), 200 μl of a suspension of infected cells per well should contain 1–2 × 10^4^ *Dictyostelium* cells to record up to 48–72 h post-infection at 25°C.

## Results

### New inhibitors of *M*. *marinum* virulence

**Fig 1:**
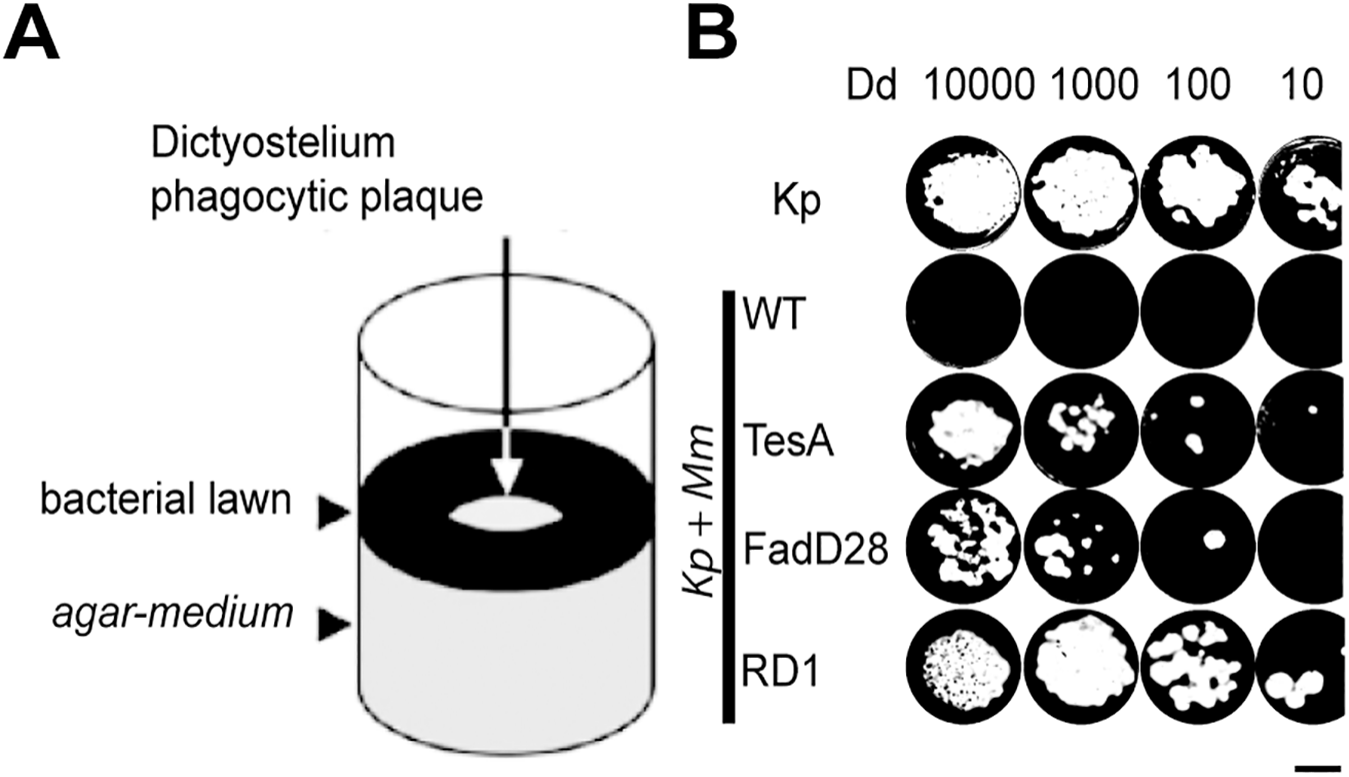
*Dictyostelium* growth on bacteria provides a measure of bacterial virulence. A. *Dictyostelium* cells deposited on a bacterial lawn formed a phagocytic plaque within 7 days. B. The ability of *Dictyostelium* to grow on a bacterial lawn was assessed by depositing 10,000, 1,000, 100 or 10 *Dictyostelium* cells on a lawn of bacteria. *Dictyostelium* grew efficiently on a lawn of non-pathogenic *Klebsiella pneumoniae* (Kp). The addition of virulent *M*. *marinum* (Kp + Mm WT) inhibited *Dictyostelium* growth. Non-virulent *M*. *marinum* mutants TesA, FadD28 and RD1 were permissive for *Dictyostelium* growth.

Growth of *Dictyostelium* on bacteria has been used as a reliable assay to measure bacterial virulence. Amoebae feed upon non-pathogenic bacteria and form phagocytic plaques in the bacterial lawn, whereas pathogenic bacteria restrict the growth of *Dictyostelium* [26]. To assess virulence of *M*. *marinum*, *Dictyostelium* cells were grown on a mixed bacterial lawn of non-virulent *K*. *pneumoniae* (for feeding of amoebae) and of virulent *M*. *marinum*, which inhibit the growth of the amoebae [19] (Fig 1). In the presence of virulent *M*. *marinum*, even 10,000 *Dictyostelium* cells were unable to clear bacteria and to form a phagocytic plaque. Three different mutant strains that were previously shown to exhibit decreased virulence were used to validate this assay: TesA mutant bacteria are defective for synthesis of major cell wall-associated lipids [19], FadD28 mutant bacteria fail to produce both PDIMs and PGLs [20] and the RD1 mutant strain lacks the RD1 virulence gene cluster [27]. In all three cases, decreased mycobacterial virulence restored the growth of *Dictyostelium* (Fig 1), indicating that mutations altering different facets of mycobacterial virulence are readily detected in this assay.

**Fig 2:**
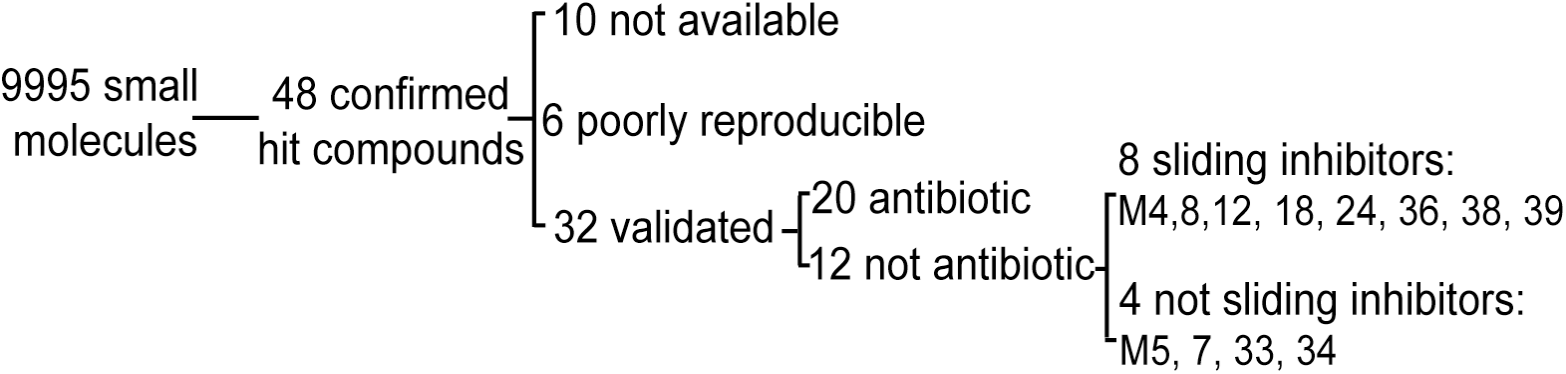
Selection and classification of *M*. *marinum* virulence inhibitors. 9,995 molecules were initially tested, from which 48 reproducibly allowed *Dictyostelium* to grow in the presence of virulent *M*. *marinum*. To validate the effect of these hit compounds, they were reordered and retested. Ten compounds were no longer commercially available. Six compounds showed a poorly reproducible effect, and 32 compounds restored *Dictyostelium* growth reproducibly. Of these 32 compounds, 20 demonstrated antibiotic activity that accounted for their effect while 12 did not and are referred to here as anti-virulence compounds. Among the latter, 8 compounds inhibited the “sliding motility” of *M*. *marinum*.

**Fig 3:**
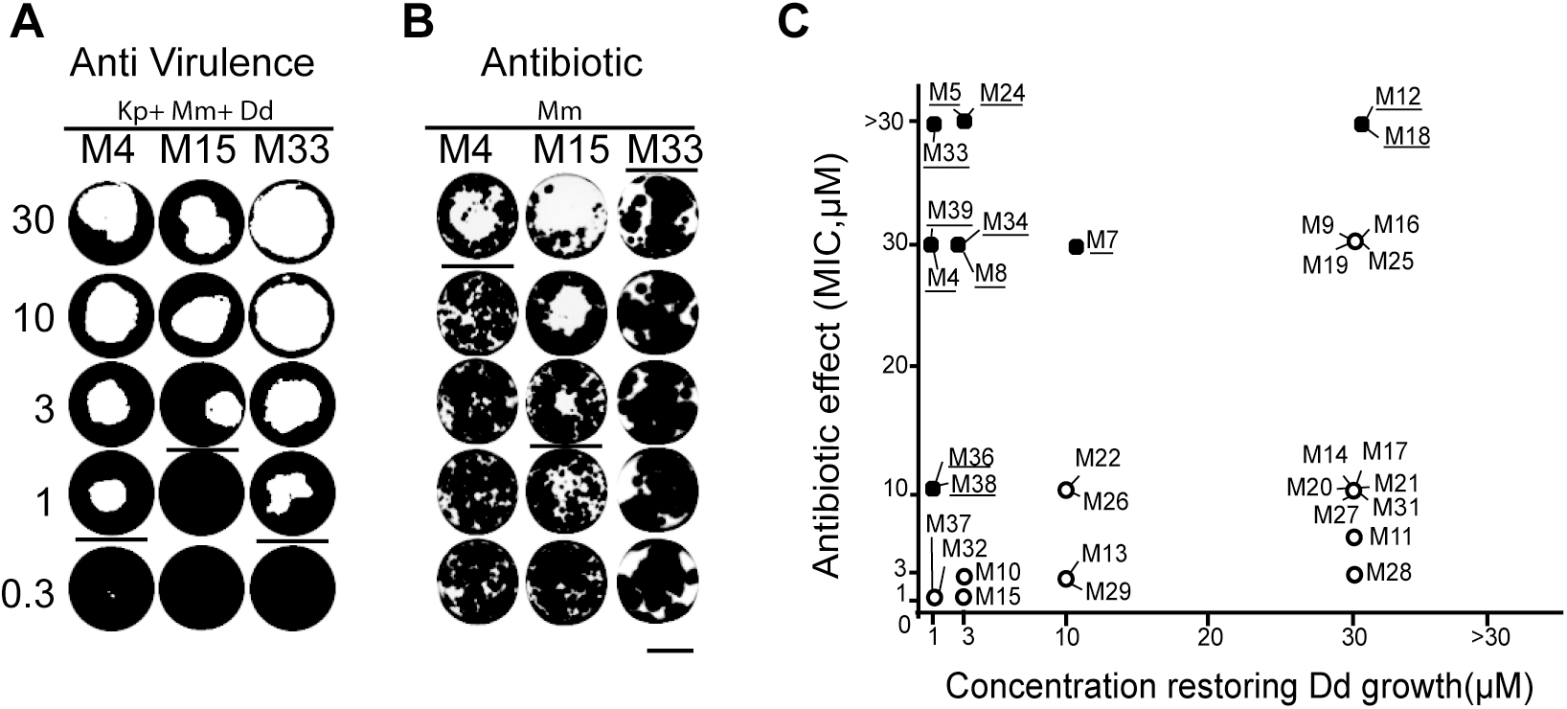
Inhibition of mycobacterial virulence and growth. A. Virulence assay. *Dictyostelium* growth in the presence of WT *M*. *marinum* and of compounds M4, M15, M33 at concentrations of 0.3 to 30 μM. The results for compounds M24 and M39 are shown in Fig 5 and Fig 6, respectively. First, each compound was added on top of SM-agar medium. Then a mixture of avirulent *Klebsiella* and *M*. *marinum* was added. Finally, 1,000 *Dictyostelium* cells were deposited in the center of the well. Within 7 days of culture at 25°C a phagocytosis plaque became visible when mycobacterial virulence was inhibited. In the examples shown, the minimal concentration inhibiting virulence was 1 μM for M4 and M33 and 3 μM for M15. B. Antibiotic assay. Compounds were added to 7H11 medium in each well at the indicated concentration, then 1,000 *M*. *marinum* bacteria were deposited in the well. Growth of mycobacteria was visible after 6 days at 30°C. Compound M33 did not exhibit any antibiotic effect, M15 inhibited bacterial growth at a concentration of 3 μM or higher, and M4 inhibited growth of bacteria at 30 μM. C. Comparison of virulence and growth MIC. For each compound tested, the minimum concentration at which it inhibited mycobacterial virulence and mycobacterial growth are indicated. Compounds inhibiting *M*. *marinum* virulence at concentrations at which no antibiotic effect was detectable are underlined and marked with full circles and were selected for further analysis.

We then tested a total of 9,995 compounds at a concentration of 30 μM for their ability to restore growth of *Dictyostelium* cells in this assay. For this, several libraries of chemically diverse compounds were used (Table 1). Compounds inhibiting growth of *K*. *pneumoniae* were not selected for further analysis, a procedure that eliminated 20 antibiotics. The initial hits (316) were then retested, leading finally to the identification of 48 hit compounds (Table 1 and Fig 2). This strict selection procedure selected only compounds with strong and reproducible effects, but it is likely that is also eliminated mistakenly some active compounds. Thirty-two of the 48 hit compounds were commercially available and reproducibly restored growth of *Dictyostelium* in the presence of *M*. *marinum*. For each of these validated hits, the minimal concentration restoring *Dictyostelium* growth (Virulence Minimal Inhibitory Concentration) was determined (Fig 3).

A compound restoring growth of *Dictyostelium* in the presence of virulent *M*. *marinum* could in principle act either by selectively inhibiting growth of *M*. *marinum* on the plate (without inhibiting *Klebsiella* growth), or by decreasing virulence of *M*. *marinum*. In this manuscript, we refer to the first possibility as an antibiotic compound, and to the second as an anti-virulence compound. To determine more precisely the mode of action of each compound, we tested its ability to directly inhibit growth of *M*. *marinum* and growth of *Klebsiella*. As expected, none of the selected compounds inhibited growth of *Klebsiella*. On the contrary, 20 compounds inhibited *M*. *marinum* growth at a concentration similar to or smaller than that required to restore growth of *Dictyostelium* in the presence of *M*. *marinum* (Fig 3) (S1 Table). For a few selected compounds (M5, M24, M33, M39) it was verified further that they did not inhibit bacterial growth in a liquid culture at a concentration of 10μM (S1 Fig). These 20 compounds are thus expected to act mostly by specifically inhibiting mycobacterial growth, and were not investigated further in this study. According to this selection, 12 compounds were finally selected as putative inhibitors of bacterial virulence: M4, 5, 7, 8, 12, 18, 24, 33, 34, 36, 38 and 39 (Table 2).

**Table 2:** Selected mycobacterial virulence inhibitors.

Code	Reference	Mw
**M4**	CAS: 12542-36-8	578.6
**M5**	CAS: 127-47-9	328.49
**M7**	CAS: 2752-65-0	628.75
**M8**	ZINC19940158	432.37
**M12**	ZINC13799740	358.39
**M18**	ZINC00315276	291.39
**M24**	ZINC09007974	324.38
**M33**	ZINC12366919	455.76
**M34**	ZINC01040827	395.87
**M36**	ZINC00069763	330.34
**M38**	ZINC01047885	400.33
**M39**	ZINC01035926	476.43

### Inhibitors of *M*. *marinum* sliding motility

**Fig 4:**
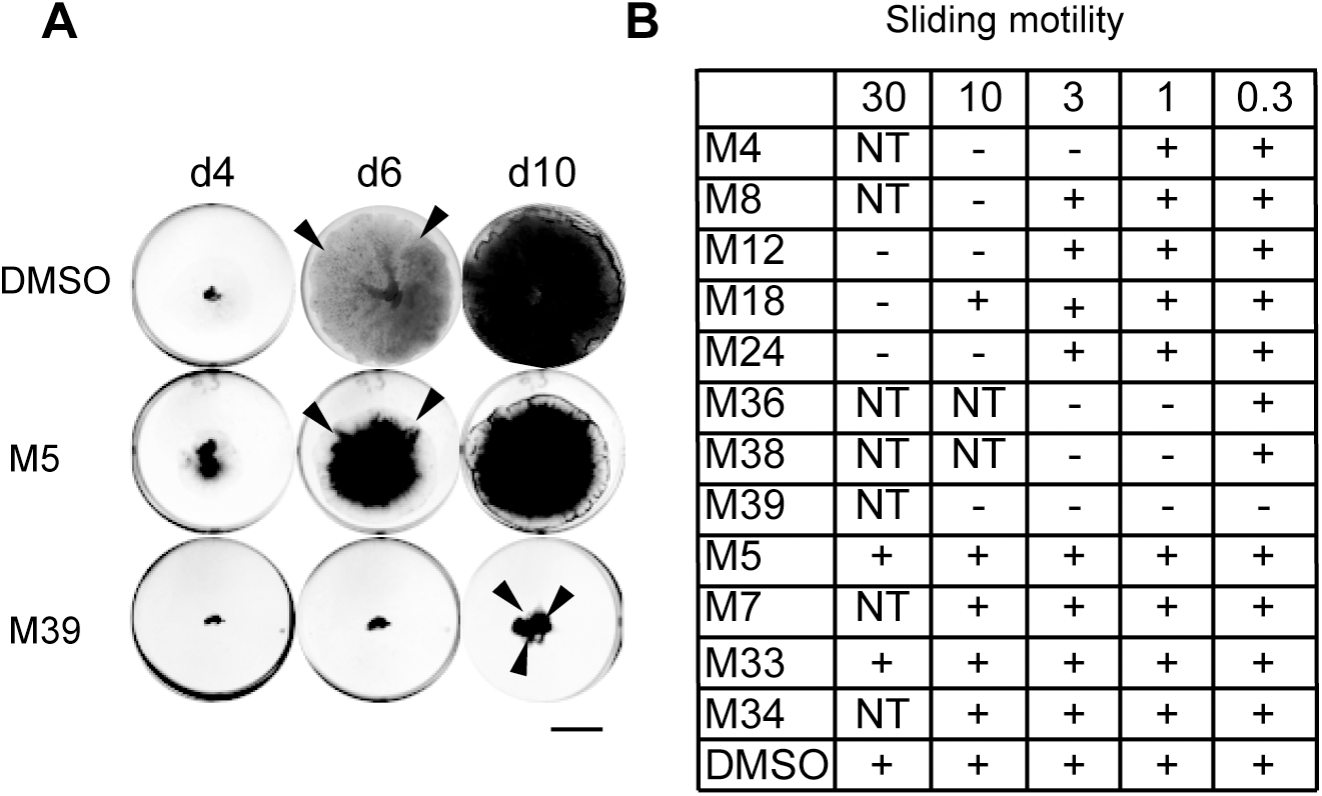
Inhibition of *M*. *marinum* sliding motility. A. Sliding motility of *M*. *marinum* was determined in the presence of compounds M39, M5 (0.3 μM each) or DMSO. Compounds were added in the center of each well containing 7H9 medium supplemented with 0.3% agarose, then mycobacteria were inoculated in the center and allowed to grow for 10 days at 30°C. The borders of the bacterial colony are indicated with black arrows. After 6 to 10 days, mycobacteria spread over the whole surface of the well. Spreading was inhibited by M39, but not by M5. Bar: 1 cm. B. Effect of each compound on sliding motility; (+) or (-) indicates if sliding motility occurred or not, respectively. For each compound, sliding motility could only be tested at concentrations where bacterial growth was not inhibited (NT: not tested).

“Sliding motility” describes the spreading of a bacterial colony as it grows on semi-solid agar media [23]. Defects in sliding motility were notably observed for *M*. *marinum* mutants exhibiting decreased virulence [28]. In order to examine whether virulence inhibitors perturbed sliding motility, isolated colonies of *M*. *marinum* were inoculated and bacteria were allowed to grow for 2 weeks at 30°C in the presence of anti-virulence compounds. Note that the effect of a compound on sliding motility could only be determined in this assay at concentrations where bacterial growth was not inhibited. Of 12 compounds tested, a total of 8 compounds affected sliding motility (M4, 8, 12, 18, 24, 36, 38 and 39). Among these compounds, M39 was most efficient at inhibiting *M*. *marinum* virulence (MIC 0.3μM) and sliding motility (MIC 0.3μM) (Fig 4). On the contrary, compounds M5, 7, 33 and 34 did not affect sliding motility (Fig 4).

The coincidental observation that a compound inhibits both bacterial virulence and sliding motility does not in itself establish a causal relationship between these two effects. To analyze the putative link between inhibition of sliding motility and inhibition of bacterial virulence, we focused on two compounds (M39 and M24), and analyzed how their effects would be affected by modification of the original compounds.

**Fig 5:**
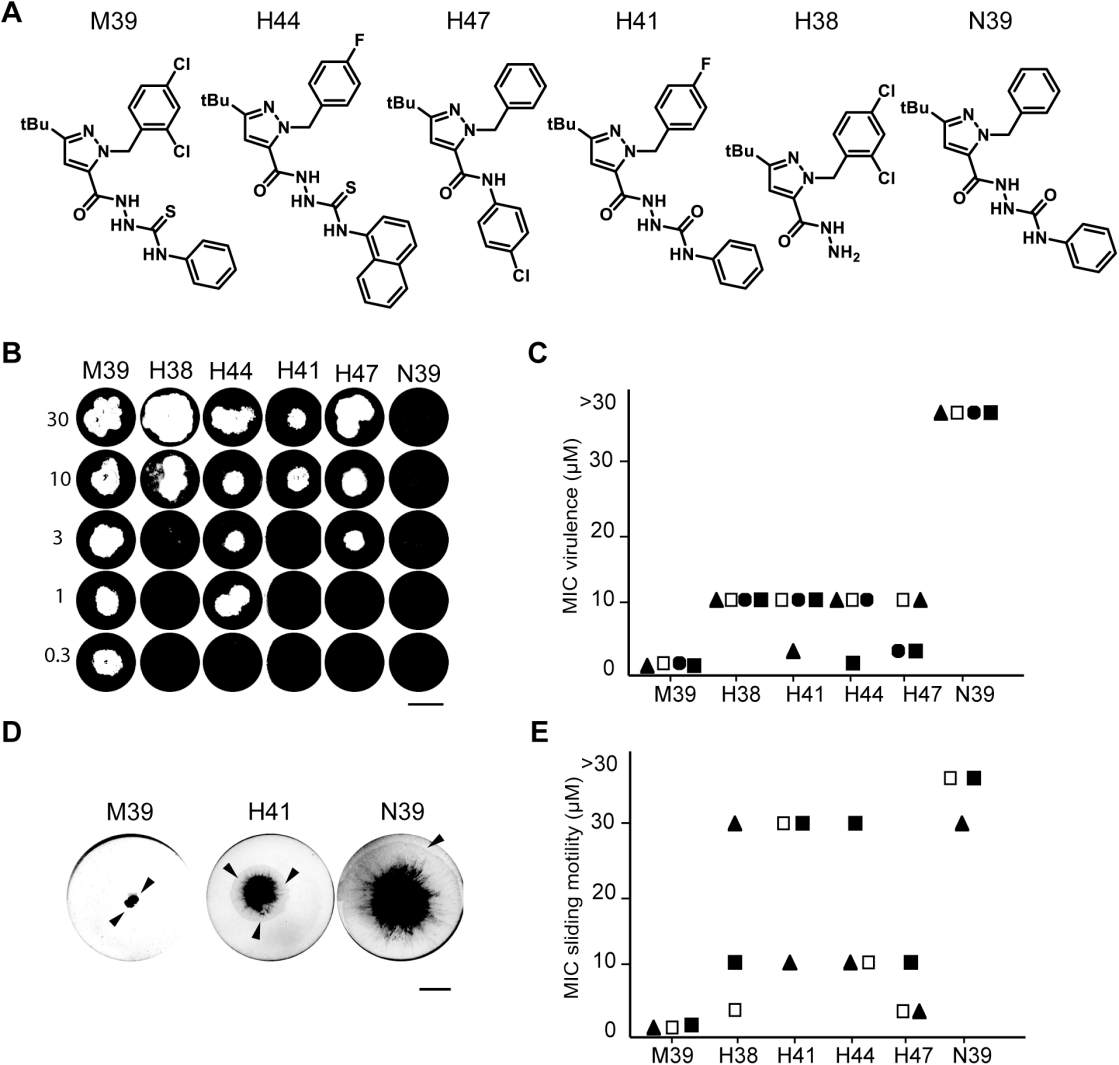
Chemical modifications of M39 concomitantly alter inhibition of *M*. *marinum* virulence and sliding motility. A. Chemical structure of M39 and its variants H38, H41, H44, H47 and N39. B. Virulence of *M*. *marinum* was assessed in the presence of increasing concentrations of each compound (0.3 to 30 μM). M39 inhibited *M*. *marinum* virulence at all concentrations down to 0.3μM, analog N39 showed no effect at all concentrations tested. Scale bar: 1 cm. C. For each compound, the minimal concentration inhibiting *M*. *marinum* virulence was determined in four independent experiments and is indicated with a different symbol for each experiment (the experiment shown in B is represented with full squares). D. Sliding motility of *M*. *marinum* in the presence of compounds M39, H41 and N39 (10μM) was analyzed as described in Fig 4. Sliding motility was inhibited efficiently by M39, partially by H41, and not at all by N39. E. For each compound, the minimal concentration inhibiting *M*. *marinum* sliding motility was determined in three independent experiments and is indicated.

**Fig 6:**
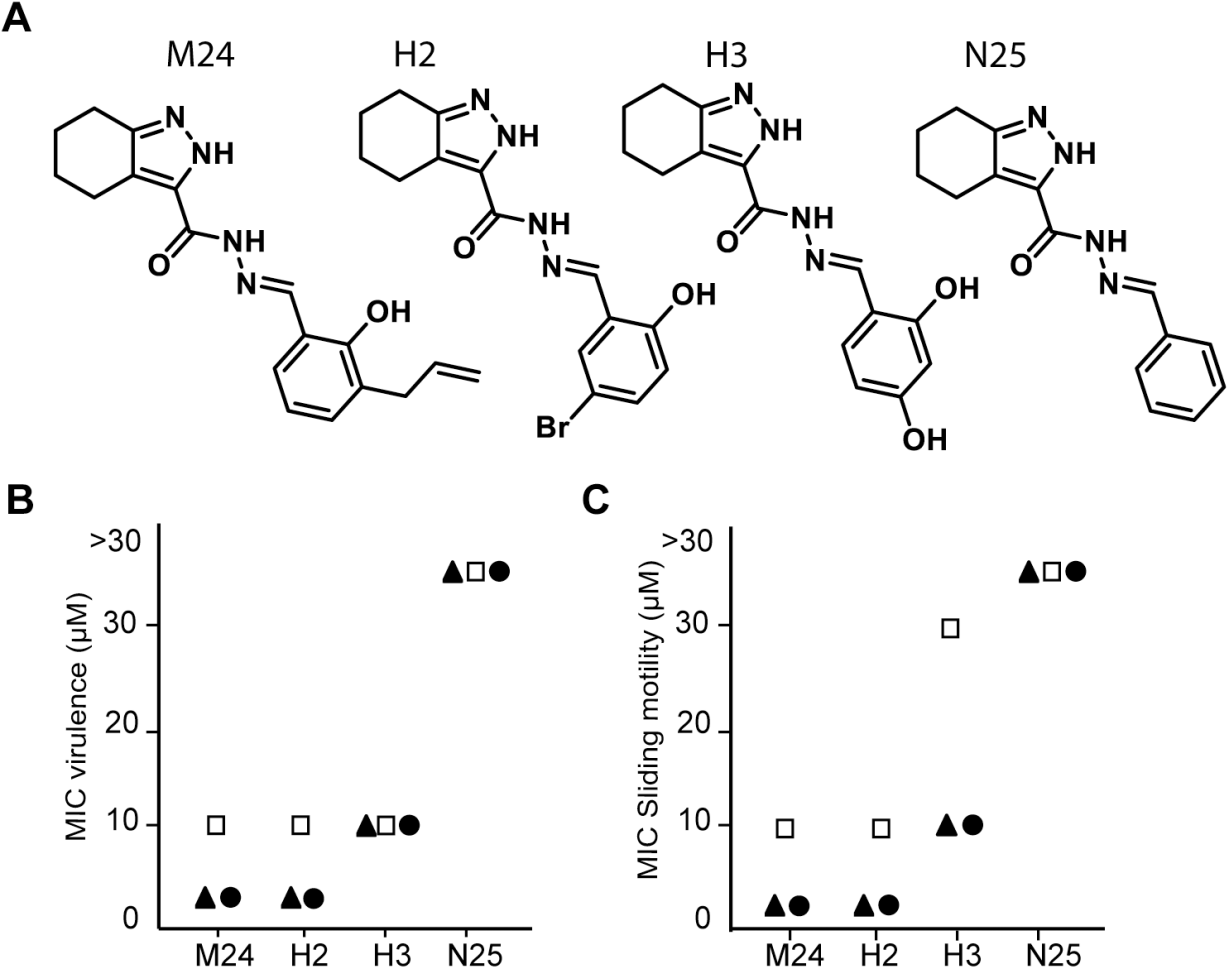
Chemical modifications of M24 concomitantly alter the compound’s activity on *M*. *marinum* virulence and sliding motility. As described in Fig 5, analogs of M24 (A) were tested for their ability to inhibit *M*. *marinum* virulence (B) and sliding motility (C). The results of three independent experiments are indicated each with a different symbol.

Five structural variants of M39 were analyzed (Fig 5). H38, H41, H44 and H47 retained the ability to inhibit bacterial virulence, albeit at concentrations slightly higher than M39 (Fig 5). N39 did not inhibit bacterial virulence at any concentration, although its structure is closely related to that of M39 (Fig 5). These five compounds were then tested for their ability to inhibit sliding motility of *M*. *marinum*. Qualitatively, at a concentration of 10 μM H41 partially retained the ability to inhibit sliding motility, while N39 was inactive (Fig 5). Quantitatively, in three independent experiments, H38, H41, H44 and H47 retained the ability to inhibit sliding motility, but at a higher concentration than M39. N39 did not reproducibly inhibit sliding motility. Thus, alterations in the M39 structure concomitantly affected its ability to inhibit *M*. *marinum* virulence and sliding motility. Similar observations were made when structural variants of compound M24 (Fig 6) were analyzed: the compounds H2 and H3 still inhibited bacterial virulence, but N25 did not (Fig 6). Concomitantly, H2 and H3 inhibited sliding motility, but N25 did not (Fig 6).

In summary, for both compounds M24 and M39, chemical modifications that decreased the ability to inhibit *M*. *marinum* sliding motility also decreased to a similar extent the effect of the compound on bacterial virulence. One possible interpretation of these results is that the primary effect of M24 and M39 is to inhibit sliding motility, and that this then results in a decrease in bacterial virulence.

### Alterations of the *M*. *marinum* envelope

**Fig 7:**
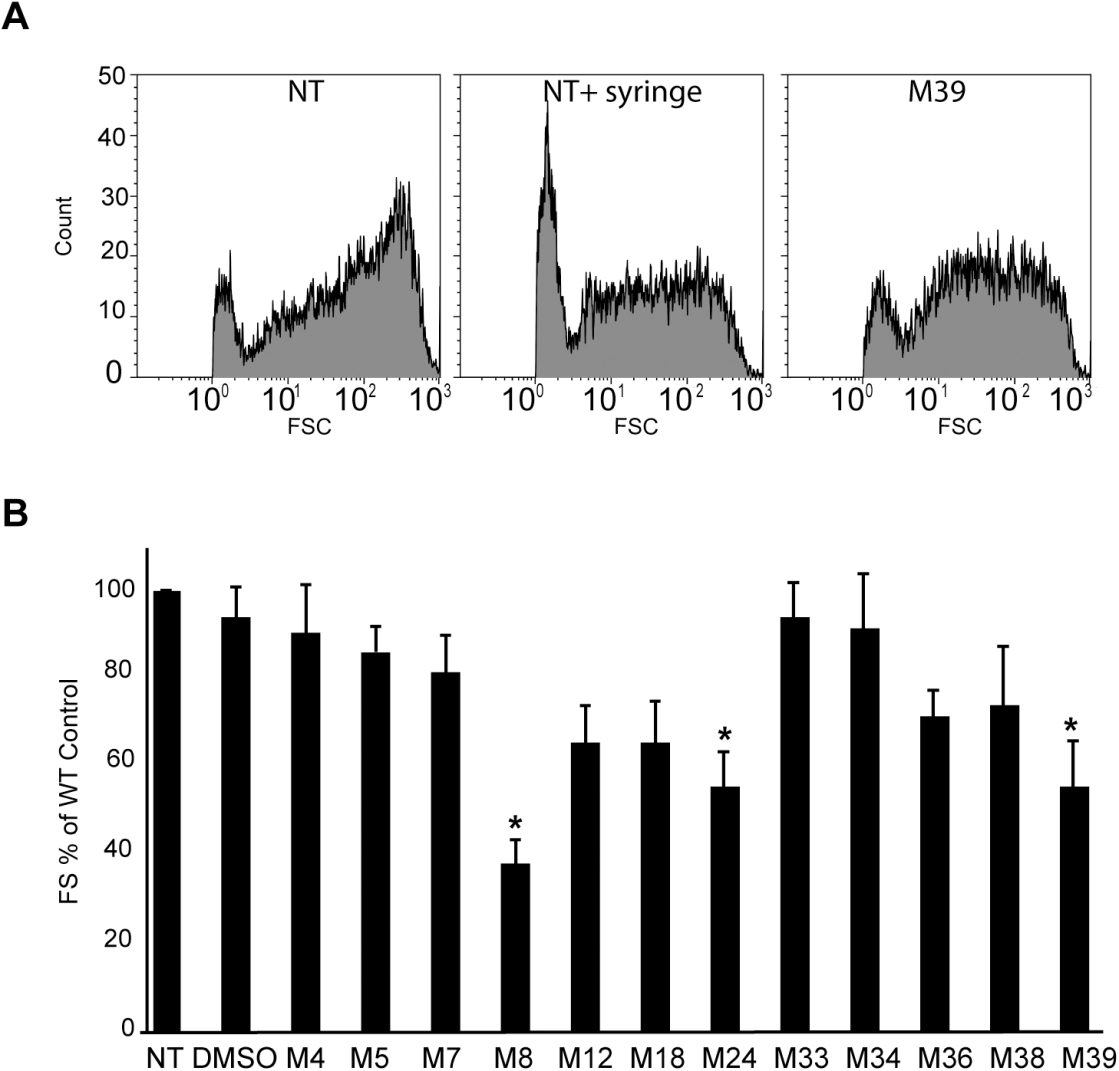
Effect of anti-virulence compounds on mycobacterial aggregation. In order to assess mycobacterial aggregation, cultures were analyzed by flow cytometry and the forward scatter (FSC) of bacterial aggregates was recorded. (A) Untreated cells (NT) aggregated readily, and as expected, repeated passage through a needle (NT + needle) reduced aggregation. Cultivation in the presence of compound M39 (10 μM) reduced the degree of aggregation. (B) In five independent experiments, the mean FSC of *M*. *marinum* cultures was measured, and expressed as a percentage of FSC measured in an untreated culture. Three compounds (M8, M24, M39) significantly inhibited mycobacterial aggregation (one-way analysis of variance: p = 0.0002; *: post-hoc Tukey-Kramer p<0.05).

Modifications in sliding motility can be caused by alterations in the properties of the mycobacterial envelope, and several mutations affecting the synthesis of envelope constituents indeed also affect sliding motility [29]. This led us to test the effect of selected compounds on other properties linked to the *M*. *marinum* envelope: bacterial aggregation and permeability. To assess mycobacterial aggregation, we grew mycobacteria in the presence of 10 μM of each compound for 48 h, then analyzed the size of bacterial aggregates using flow cytometry by measuring the forward scatter of bacterial aggregates (Fig 7). Mycobacteria formed large aggregates in untreated cultures, which as expected disassembled partly when the bacterial culture was homogenized by repeated passing through a syringe needle (Fig 7). Exposure to compound M39 reduced the number of aggregates with higher sizes (Fig 7). Of 12 compounds tested, 3 molecules (M8, M24 and M39) significantly decreased bacterial aggregation in this assay (Fig 7).

**Fig 8:**
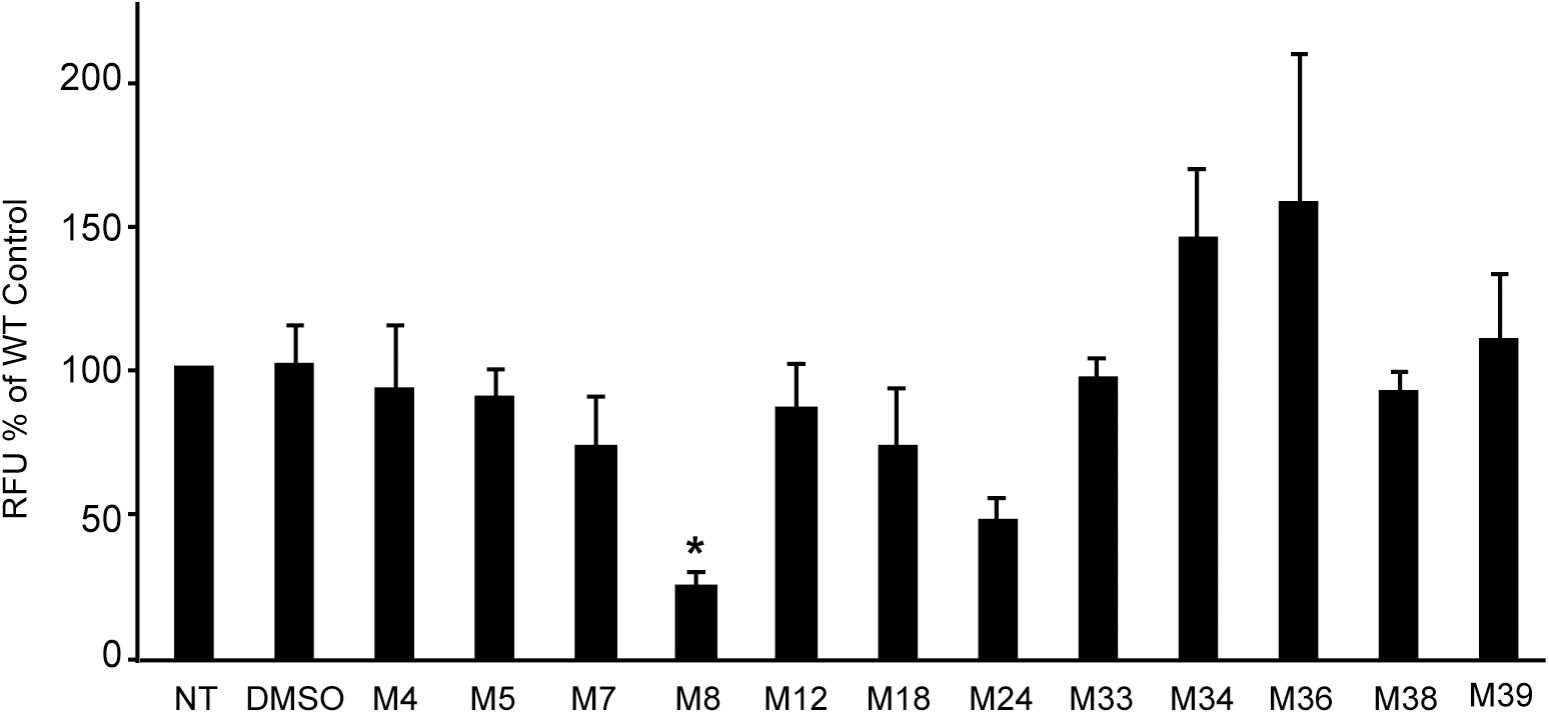
Effect of anti-virulence compounds on mycobacterial cell wall permeability. Mycobacteria were grown in the presence of each compound (10 μM), incubated for 20 min in the presence of 6μM ethidium bromide, and analyzed by flow cytometry. Analysis of fluorescence was performed on bacteria with very similar aggregate size. The average and SEM of five or six independent experiments is presented. One compound (M8) significantly inhibited bacterial permeability (one-way analysis of variance: p = 0.0002; *: post-hoc Tukey-Kramer p<0.05).

Bacterial permeability was tested in the same experiments by incubating bacteria in the presence of ethidium bromide and then measuring the amount of dye penetrating the cells (Fig 8). One compound (M8) significantly decreased bacterial permeability in this assay. The variations observed upon exposure to other compounds were not statistically significant.

Finally, we tested directly whether the concentration of phenolic glycolipids (PGL) and phthiocerol dimycocerosates (PDIM) in the *M*. *marinum* envelope were affected upon treatment with anti-virulence compounds. The amounts of PGL and PDIM were measured as described previously using two-dimension thin layer chromatography (2D-TLC) [30, 31]. After treatment for 48 h at 10 μM, none of the compounds tested visibly decreased the amount of PGL and PDIM (S2 Fig). It seems likely that they affect some other (unidentified) element of the complex mycobacterial wall.

### Antivirulence compounds inhibit intracellular replication of *M*. *marinum* within *Dictyostelium*

**Fig 9:**
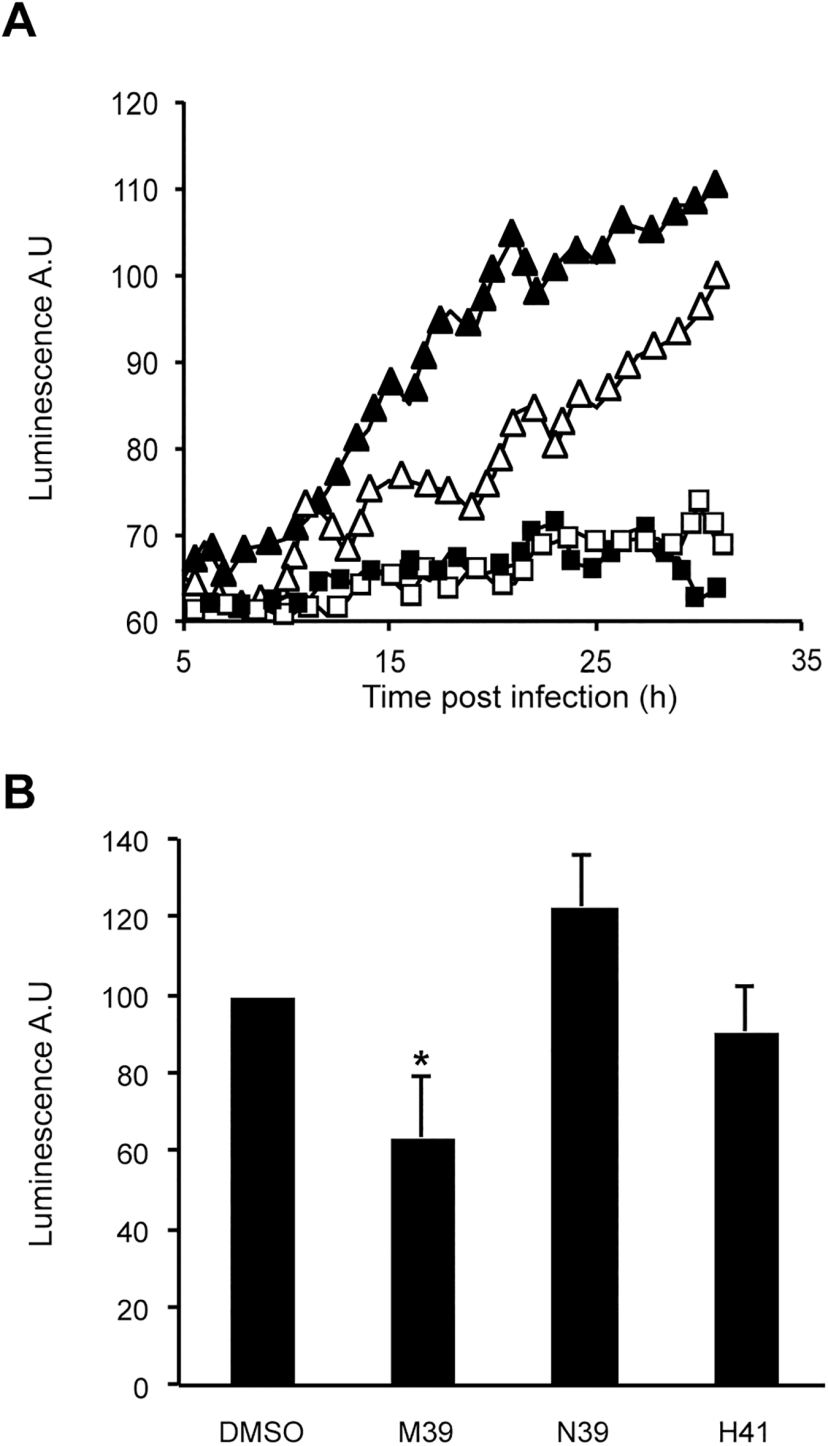
M39 inhibits intracellular replication of *M*. *marinum*. A. *Dictyostelium* cells were infected at time 0 with luminescent *M*. *marinum* grown for 24 h in the presence of DMSO (empty triangle), M39 (black square), H41 (empty square) or N39 (full triangle) (10 μM). Intracellular replication was assessed by measuring the increase in intracellular luminescence. B. The experiment described in A was performed three times independently and the level of luminescence at 24 h was recorded. The average and SEM are indicated. *: significantly different from NT (student’s t-test; p<0.05).

One hallmark of an anti-virulence compound is that it would be expected to inhibit intracellular replication of mycobacteria. In order to examine directly whether selected compounds inhibit intracellular replication of *M*. *marinum*, we focused on compound M39 and two of its variants, one active (H41), and one inactive (N39). Mycobacteria expressing bacterial luciferase plasmid were incubated with compounds (10 μM) for 24 h, and used to infect *Dictyostelium* cells in the continued presence of compounds. Intracellular replication of *M*. *marinum* was followed by measuring the increase in luminescence (Fig 9). M39 as well as its active variant H41 inhibited the intracellular replication of *M*. *marinum* (Fig 9). As expected, the inactive variant N39 did not inhibit intracellular bacterial replication (Fig 9). These data confirm that the screening strategy employed in this study successfully identified compounds that decrease virulence of mycobacteria and, at least in the case of the M39 series of analogs, can effectively inhibit their intracellular replication.

## Discussion

In this study, we used a host-based assay to measure the pathogenicity of *M*. *marinum* and to isolate small molecules inhibiting virulence of these mycobacteria. Since this screen was not intentionally biased towards inhibitors of a specific virulence mechanism, any facet of mycobacterial physiology implicated in virulence represented a potential target. We identified 12 small molecules that inhibited virulence of *M*. *marinum* at concentrations that did not inhibit its growth in broth, and thus represent *bona fide* virulence inhibitors, distinct from classical antibiotics. A first classification of the selected compounds was performed, based on a few phenotypic assays, and the results obtained suggest that the selected compounds may have very different modes of action. Eight of the 12 compounds inhibited at least to some extent *M*. *marinum* sliding motility, three of these (M8, M24, M39) also decreased formation of bacterial aggregates, and one (M8) decreased in addition membrane permeability of bacteria. For two of these compounds (M39 and M24), analysis of structural variants revealed that the effect on sliding motility was decreased or lost concomitantly with the effect on bacterial virulence. To our knowledge, this is the first study identifying inhibitors of mycobacterial sliding motility.

Overall, it seems likely that the eight compounds inhibiting sliding motility of *M*. *marinum* act mostly by altering the complex composition or organization of the mycobacterial cell wall, which is the main determinant of sliding motility. However, other functions associated with the mycobacterial envelope (propensity to aggregate, permeability) were affected to a very variable degree by each of these compounds, suggesting that they induce different types of alterations. The analysis of mycobacterial lipids did not reveal gross alterations upon treatment with the selected compounds, and further analysis will be necessary to determine the exact biochemical modifications leading to alterations of sliding motility.

In addition to the compounds affecting mycobacterial sliding motility, several compounds (M5, M7, M33 and M34) did not exhibit any effect on the phenotype of *M*. *marinum* in the assays used here. These compounds presumably act on some other facet(s) of *M*. *marinum* physiology, not tested in this study. Alternatively, they may act primarily on the host, boosting cellular defenses against *M*. *marinum*.

In this study, we isolated inhibitors of *M*. *marinum* virulence by directly measuring the virulence of the bacteria in a *Dictyostelium* host model. Such a host-based screening has the advantage of identifying compounds that are capable of entering mycobacteria to alter their phenotype, while excluding toxic compounds that would kill host cells. Indeed, we obtained no evidence that any of the compounds isolated is toxic. This approach also allows the isolation of a variety of compounds with different modes of actions. Finally, we tested further one of the isolated compounds and showed that it affects the ability of mycobacteria to replicate in infected cells. Very few reports describe inhibitors of mycobacterial virulence. A previous screen was designed specifically to isolate inhibitors of mycobacterial protein secretion [32]. In other screens, inhibitors of selected mycobacterial targets were identified, such as inhibitors of the PhoPR regulon [33], the MycP1 protease [34], the Zmp1 metalloprotease [35], or the PtpB phosphatase [36]. We are not aware of any previously described screening that would potentially identify virtually any inhibitor of mycobacterial virulence. This may account for the fact that our screen exclusively identified new inhibitors that were aimed at least in part at a previously untargeted pathway, mycobacterial sliding motility. Further studies will be necessary to identify the exact mode of action and molecular target of each of these compounds, since many gene products are essential for sliding motility and for mycobacterial virulence in general, and thus represent possible targets for such inhibitors [20, 29, 37]. It will also be interesting to determine in other model systems (e.g. zebrafish infection) if the compounds identified in our study also inhibit mycobacterial infections. It remains to be seen whether these compounds would act on other mycobacteria, and in particular on *M*. *tuberculosis*. Besides revealing new aspects of *M*. *marinum* virulence, these studies may lead to the identification and characterization of new anti-mycobacterial compounds with therapeutic potential.

## Supporting information

S1 Table Compounds with antibiotic activity against M. marinum. (DOCX)

S1 Fig Effect of compounds M5, M24, M33 and M39 on mycobacterial growth. *M*. *marinum* bacteria were grown in 7H9 medium for 55h in the presence of DMSO, or 10μM of compounds M5, M24, M33 or M39. OD600 was measured at the indicated times (A). After 24h of growth, the colony-forming units were determined after plating dilutions of the cultures on 7H11 plates (B). No significant effect of compounds on mycobacterial growth was detected.
(TIF)

S2 Fig Two-dimension thin layer chromatography (2DTLC). Apolar lipid fractions were prepared from *M*. *marinum* (NT) grown for 24 hours in the presence of virulence inhibitors M24 and M39 (10μM), according to published procedures [1, 2]. These lipids were analyzed by two-dimensional thin layer chromatography (2D-TLC) on silica gel 60 plates (EMD Chemicals Inc). For PDIM development lipids were migrated in petroleum ether-ethyl acetate (98:2, v/v, 3 times) in the first dimension and petroleum ether-acetone (98:2, v/v) in the second dimension. The plates were sprayed with 5% molybdophosphoric acid 95% ethyl alcohol (v/v) and heated at 150°C for 15 min. For PGL development, chloroform-methanol (96:4, v/v) was used in the first dimension followed by toluene-acetone (90:10, v/v, 3 times) in the second dimension. Plates were then spread with alpha-naphtol sulfiric acid reagent and heated at 120°C for 10 min.
(TIF)
