# Supplementary material for: Inhibitors of Mycobacterium marinum virulence identified in a Dictyostelium discoideum host model
Source: PLoS One. 2017 Jul 20;12(7):e0181121. doi: 10.1371/journal.pone.0181121 (PMC5519057; doi:10.1371/journal.pone.0181121)
Supplement: S1 Table — (DOCX) [file pone.0181121.s001.docx]

**Supporting information**

**S1 Table: compounds with antibiotic activity against M. marinum.**

| **Code** | **Reference** | **Mw** | **Source** |
| --- | --- | --- | --- |
| **M9** | **ZINC19683144** | **441.32** | **Malaria box** |
| **M10** | **ZINC01162356** | **356.42** | **Malaria box** |
| **M11** | **ZINC01791925** | **470.16** | **Malaria box** |
| **M13** | **ZINC00317967** | **321.4** | **Malaria box** |
| **M14** | **ZINC03307107** | **278.36** | **Sinergia** |
| **M15** | **ZINC01580838** | **145.2** | **Sinergia** |
| **M16** | **ZINC00254142** | **338.33** | **Sinergia** |
| **M17** | **ZINC00983974** | **193.24** | **Sinergia** |
| **M19** | **ZINC16736406** | **253.46** | **Sinergia** |
| **M20** | **ZINC04688380** | **299.36** | **Sinergia** |
| **M21** | **ZINC04795898** | **328.34** | **Sinergia** |
| **M22** | **ZINC26513872** | **210.74** | **Sinergia** |
| **M25** | **AG-690/09390052** | **284.31** | **Sinergia** |
| **M26** | **ZINC00899897** | **420.46** | **Sinergia** |
| **M27** | **ZINC23709262** | **298.75** | **Sinergia** |
| **M28** | **ZINC04060816** | **255.32** | **Sinergia** |
| **M29** | **ZINC33380972** | **342.29** | **Maybridge** |
| **M31** | **ZINC00156000** | **329.47** | **Maybridge** |
| **M32** | **ZINC00634564** | **354.35** | **Maybridge** |
| **M37** | **CAS: 26159-34-2** | **252.24** | **Maybridge** |

- **S1 Fig. Effect of compounds M5, M24, M33 and M39 on mycobacterial growth.** *M. marinum* bacteria were grown in 7H9 medium for 55h in the presence of DMSO, or 10µM of compounds M5, M24, M33 or M39. OD600 was measured at the indicated times (A). After 24h of growth, the colony-forming units were determined after plating dilutions of the cultures on 7H11 plates (B). No significant effect of compounds on mycobacterial growth was detected.

**S2 Fig. Two-dimension thin layer chromatography (2DTLC)**. Apolar lipid fractions were prepared from *M. marinum* (NT) grown for 24 hours in the presence of virulence inhibitors M24 and M39 (10µM), according to published procedures [1, 2]. These lipids were analyzed by two-dimensional thin layer chromatography (2D-TLC) on silica gel 60 plates (EMD Chemicals Inc). For PDIM development lipids were migrated in petroleum ether-ethyl acetate (98:2, v/v, 3 times) in the first dimension and petroleum ether-acetone (98:2, v/v) in the second dimension. The plates were sprayed with 5% molybdophosphoric acid 95% ethyl alcohol (v/v) and heated at 150°C for 15 min. For PGL development, chloroform-methanol (96:4, v/v) was used in the first dimension followed by toluene-acetone (90:10, v/v, 3 times) in the second dimension. Plates were then spread with alpha-naphtol sulfiric acid reagent and heated at 120°C for 10 min.

1. Alexander DC, Jones JR, Tan T, Chen JM, Liu J. PimF, a mannosyltransferase of mycobacteria, is involved in the biosynthesis of phosphatidylinositol mannosides and lipoarabinomannan. The Journal of biological chemistry 2004: 279: 18824-18833.

2. Chen JM, Islam ST, Ren H, Liu J. 2007. Differential productions of lipid virulence factors among BCG vaccine strains and implications on BCG safety. Vaccine 25:8114–8122.
