# Supplementary figures and images for: Inhibitors of Mycobacterium marinum virulence identified in a Dictyostelium discoideum host model
Source: PLoS One. 2017 Jul 20;12(7):e0181121. doi: 10.1371/journal.pone.0181121 (PMC5519057; doi:10.1371/journal.pone.0181121)

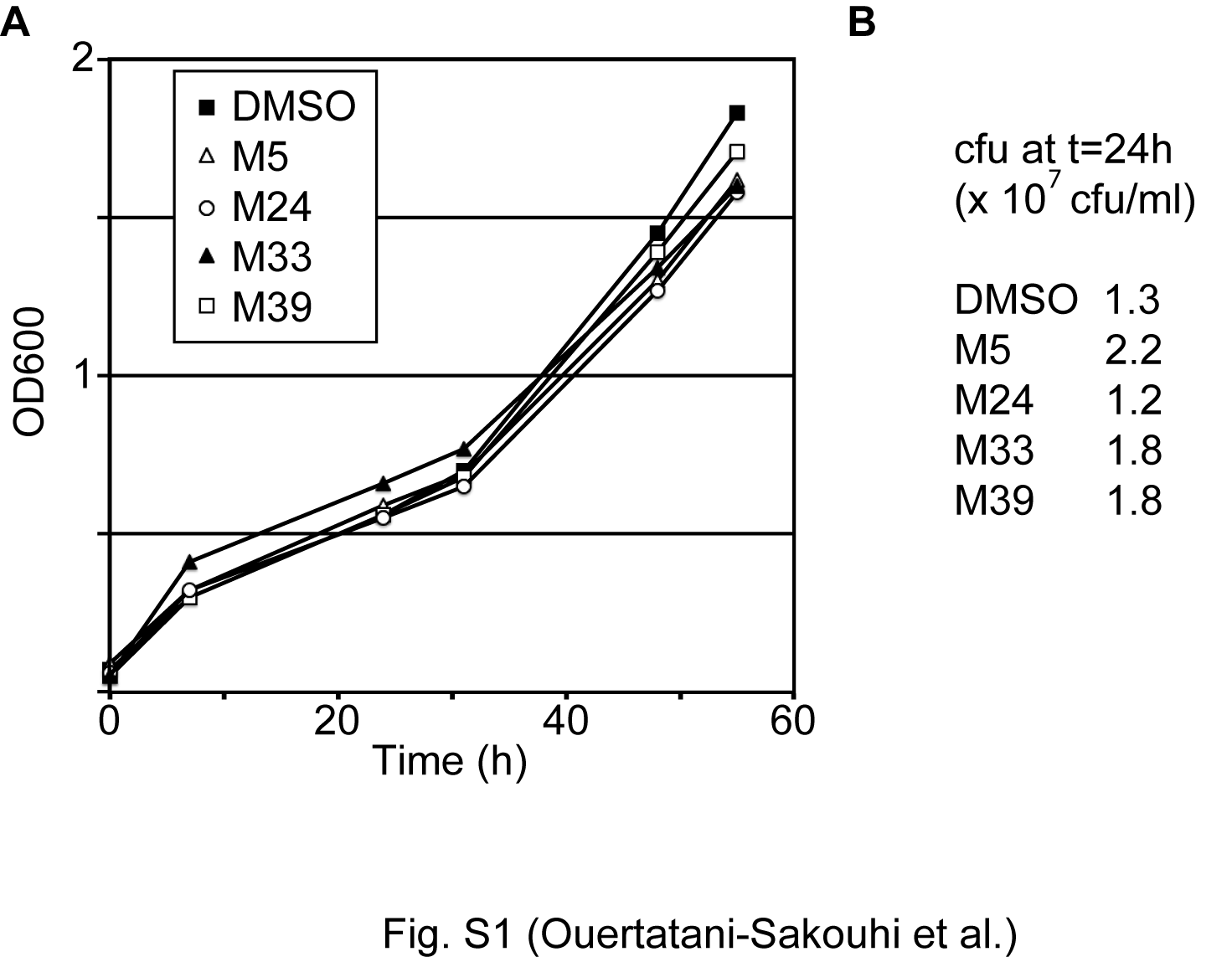

Supplement: S1 Fig — M. marinum bacteria were grown in 7H9 medium for 55h in the presence of DMSO, or 10μM of compounds M5, M24, M33 or M39. OD600 was measured at the indicated times (A). After 24h of growth, the colony-forming units were determined after plating dilutions of the cultures on 7H11 plates (B). No significant effect of compounds on mycobacterial growth was detected. (TIF) [file pone.0181121.s002.tif]

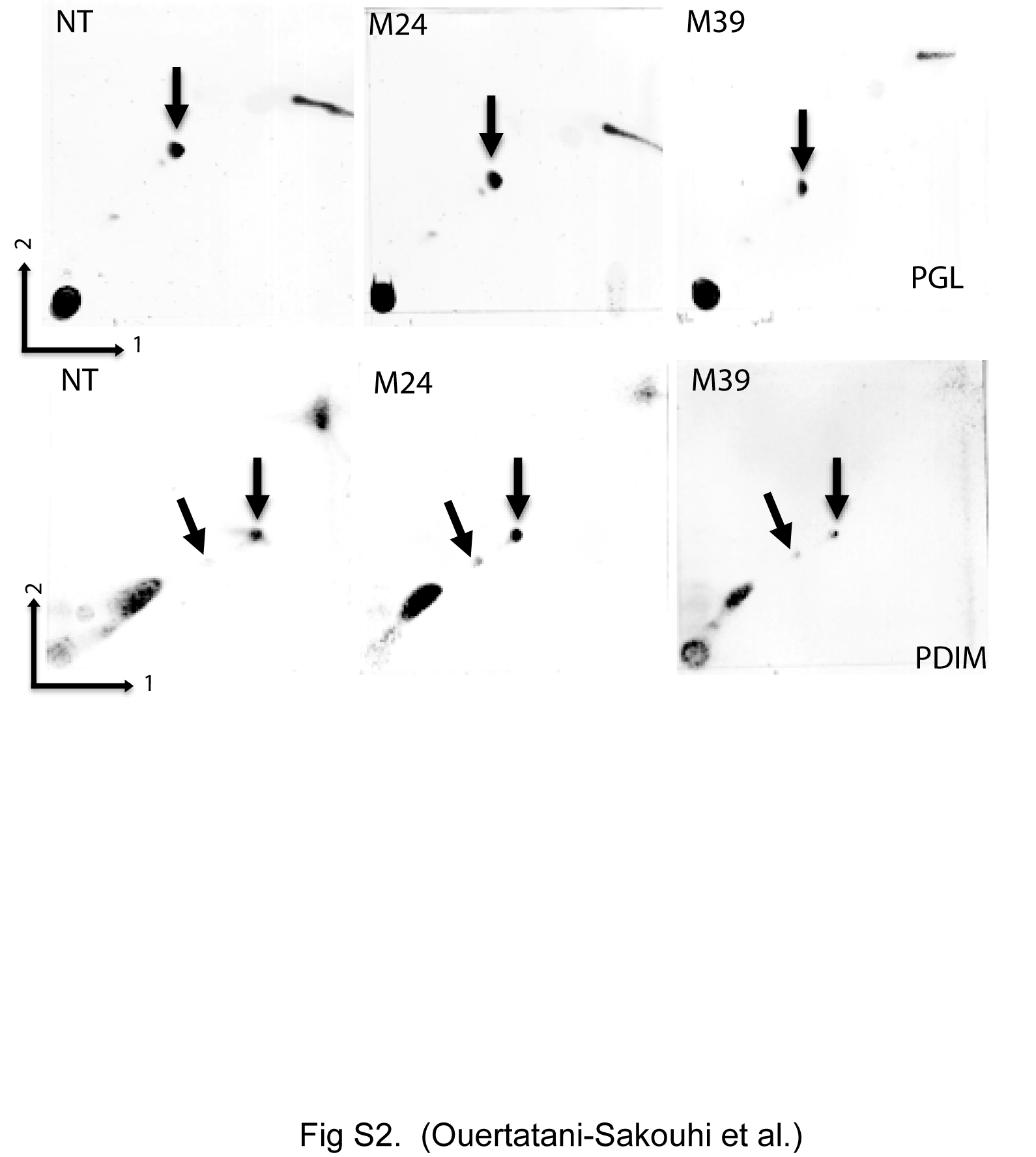

Supplement: S2 Fig — Apolar lipid fractions were prepared from M. marinum (NT) grown for 24 hours in the presence of virulence inhibitors M24 and M39 (10μM), according to published procedures [1, 2]. These lipids were analyzed by two-dimensional thin layer chromatography (2D-TLC) on silica gel 60 plates (EMD Chemicals Inc). For PDIM development lipids were migrated in petroleum ether-ethyl acetate (98:2, v/v, 3 times) in the first dimension and petroleum ether-acetone (98:2, v/v) in the second dimension. The plates were sprayed with 5% molybdophosphoric acid 95% ethyl alcohol (v/v) and heated at 150°C for 15 min. For PGL development, chloroform-methanol (96:4, v/v) was used in the first dimension followed by toluene-acetone (90:10, v/v, 3 times) in the second dimension. Plates were then spread with alpha-naphtol sulfiric acid reagent and heated at 120°C for 10 min. (TIF) [file pone.0181121.s003.tif]
